# Way to Go: Identifying Routes for Walkers and Cyclists to Avoid Air Pollutants

**DOI:** 10.1289/ehp.125-A71

**Published:** 2017-03-31

**Authors:** Carol Potera

**Affiliations:** Carol Potera, based in Montana, also writes for *Microbe*, *Genetic Engineering News*, and the *American Journal of Nursing*.

Exposures to air pollutants may offset a portion of the health benefits of walking and bicycling in cities.^1^ However, taking a detour just a block or two away from the busiest streets and roads “can make a big difference in your exposure,” says Steve Hankey, an assistant professor at Virginia Polytechnic Institute and State University and coauthor of a new study in *EHP*.^2^

**Figure:**
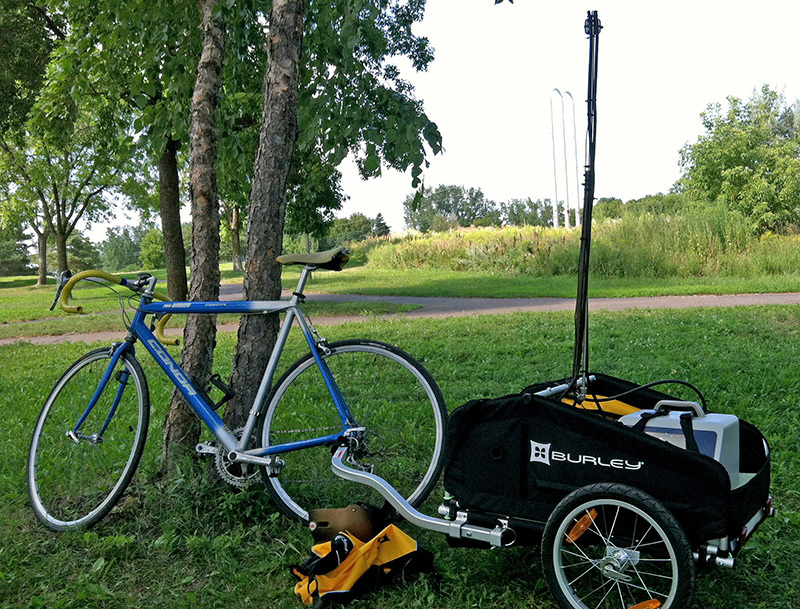
Over the course of the study, coauthor Steve Hankey covered about 1,000 miles on his bike, pulling a mobile air sampler through the streets of Minneapolis. © Steve Hankey

For every street block in Minneapolis, Minnesota—13,604 in all—Hankey and his colleagues modeled the exposure of pedestrians and cyclists to particulate air pollution during the afternoon rush hour (4:00–6:00 p.m.). Their models of pedestrian and bicycle traffic and of air quality showed that 3–7% of city blocks were what they called “sour spots,” with high levels of air pollution and high numbers of walkers and cyclists. These sour spots occurred downtown around retail stores, in the business district, and along main traffic arteries.

In contrast, 2–3% of blocks were “sweet spots,” with high rates of walking and cycling but low air pollution. Sweet spots were mostly close to the city center (and thus still walkable) but just outside of the actual downtown area (and thus had lower levels of vehicular pollution). The researchers estimate that shifting cyclists and pedestrians during rush hour from high-traffic roads onto low-traffic roads just one block away could decrease these individuals’ exposure concentrations of ultrafine particulate matter by 11%, black carbon by 19%, and fine particulate matter by 3%.^2^

“There was a high spatial mismatch between where people walked and biked and where pollution was high,” says Hankey. For example, 49% of walking and 29% of cycling in Minneapolis occurred on high-traffic, polluted streets.^2^ Hankey suggests that people living or working in cities likely could find cleaner air by walking or biking in less-trafficked areas.

The modeling results suggest strategies that city planners might use to redesign cycling and vehicular traffic flows as a way to improve public health. Practical solutions could include the establishment of bike-friendly corridors by adding speed bumps or creating one-way roads. Alternatively, bicycle routes could be relocated onto low-traffic roads, and bus traffic along popular walking routes could be shifted to corridors directly adjacent to those routes.

Hankey was a graduate student at the University of Minnesota at the time of the study. In 2012, on approximately 40 runs between late August and the end of October, he rode a bicycle pulling a trailer with instruments that measured particulate air pollutant levels. He rode around Minneapolis on three 20-mile routes that covered different road types and land uses. The data he collected were used to build a statistical model to estimate air quality block by block across the city.

“We were testing how mobile sampling by bike compares to traditional fixed-site monitoring,” says Hankey. As their name suggests, fixed-site monitors detect air quality only in the area immediately surrounding them. In contrast, the mobile sampling method used in the current study detects small changes in concentrations of pollutants all along routes where people actually walk and bike.

Hankey plans to collect more data on air pollution and numbers of pedestrians and cyclists throughout the entire day, rather than just during afternoon rush hour. “I want to scale up … to compare cities nationwide and provide more useful information for all Americans,” he says.

“[Hankey’s] novel study provides important new insights in population-level spatial patterns of exposure to air pollution during active travel that may be important for planning low-exposure cities that are overall health protective,” says Mark Nieuwenhuijsen, director of Air Pollution and Urban Environment at ISGlobal in Barcelona, Spain. In addition to shifting active travel away from major roads, Nieuwenhuijsen, who was not involved in the work, says a more sensible approach would be to reduce car use and try to create car-free zones or even car-free cities.^3^ He says, “This would reduce air pollution, noise, heat island effects, and sedentary behavior, and increase green space.”
